# Decreased expression of 17β-hydroxysteroid dehydrogenase type 1 is associated with DNA hypermethylation in colorectal cancer located in the proximal colon

**DOI:** 10.1186/1471-2407-11-522

**Published:** 2011-12-19

**Authors:** Agnieszka Anna Rawłuszko, Karolina Horbacka, Piotr Krokowicz, Paweł Piotr Jagodziński

**Affiliations:** 1 Department of Biochemistry and Molecular Biology, Poznań University of Medical Sciences, Poznań Poland; 2 Department of General and Colorectal Surgery, Poznań University of Medical Sciences, Poznań Poland

## Abstract

**Background:**

The importance of 17β-estradiol (E2) in the prevention of large bowel tumorigenesis has been shown in many epidemiological studies. Extragonadal E2 may form by the aromatase pathway from androstenedione or the sulfatase pathway from estrone (E1) sulfate followed by E1 reduction to E2 by 17-β-hydroxysteroid dehydrogenase (HSD17B1), so *HSD17B1 *gene expression may play an important role in the production of E2 in peripheral tissue, including the colon.

**Methods:**

*HSD17B1 *expression was analyzed in colorectal cancer cell lines (HT29, SW707) and primary colonic adenocarcinoma tissues collected from fifty two patients who underwent radical colon surgical resection. Histopathologically unchanged colonic mucosa located at least 10-20 cm away from the cancerous lesions was obtained from the same patients. Expression level of *HSD17B1 *using quantitative PCR and western blot were evaluated. DNA methylation level in the 5' flanking region of *HSD17B1 *CpG rich region was assessed using bisulfite DNA sequencing and HRM analysis. The influence of DNA methylation on HSD17B1 expression was further evaluated by ChIP analysis in HT29 and SW707 cell lines. The conversion of estrone (E1) in to E2 was determined by electrochemiluminescence method.

**Results:**

We found a significant decrease in HSD17B1 transcript (*p *= 0.0016) and protein (*p *= 0.0028) levels in colorectal cancer (CRC) from the proximal but not distal colon and rectum. This reduced *HSD17B1 *expression was associated with significantly increased DNA methylation (*p *= 0.003) in the CpG rich region located in the 5' flanking sequence of the *HSD17B1 *gene in CRC in the proximal but not distal colon and rectum. We also showed that 5-dAzaC induced demethylation of the 5' flanking region of *HSD17B1*, leading to increased occupation of the promoter by Polymerase II, and increased transcript and protein levels in HT29 and SW707 CRC cells, which contributed to the increase in E2 formation.

**Conclusions:**

Our results showed that reduced *HSD17B1 *expression can be associated with DNA methylation in the 5' flanking region of *HSD17B1 *in CRC from the proximal colon.

## Background

Colorectal cancer (CRC) is the third in the United States [1] and second in Europe [2] cause of malignant disease deaths among adults. Population based studies have shown significant gender differences in CRC incidence and mortality [3]. CRC occurrence is more frequent among men than in pre-menopausal women, which suggests a protective role of 17-β-estradiol (E2) in the development of this cancer [3-5]. Moreover, many case-control and cohort studies showed an inverse relationship between the risk of CRC incidence and the use of hormone replacement therapy by post-menopausal women [6,7]. Although E2 is mainly biosynthesized by the ovaries, this hormone can also be produced in peripheral tissues in both genders [8]. Extragonadal E2 can be formed by the aromatase pathway from the androstenedione or the sulfatase pathway from estrone (E1) sulfate, followed by E1 reduction to E2 by 17-β-hydroxysteroid dehydrogenase (HSD17B1) [8,9]. Therefore, *HSD17B1 *gene expression may play an important role in the production of E2 in peripheral tissue. *HSD17B1 *transcription may start from distal or proximal promoters, forming, respectively, the long (2.3-kb) or short (1.3-kb) transcript, but the short mRNA is thought to be translated into the HSD17B1 protein (Additional file 1) [10-13].

Epigenetics involve variable usage of DNA due to chromatin modifications without disturbances in the DNA sequence [14]. The low level of DNA methylation within 5'-CpG-3' dinucleotides may relate to the activation of transcription or chromosomal instability [14,15]. It has been shown that, similarly to genetic mutations, hypermethylation or hypomethylation of gene promoters may change the expression of cancer related genes in different malignancies, including CRC [14]. DNA methylation is carried out by DNA methyltransferases (DNMTs), and increased levels of some DNMTs account for transcriptional silencing of cancer protective genes [15].

We studied HSD17B1 transcript and protein levels in primary cancerous tissue and histopathologically unchanged colorectal tissue from the same fifty two patients with CRC. We also evaluated the effect of 5-Aza-2'-deoxycytidine (5-dAzaC), a DNMTs inhibitor, on HSD17B1 transcript and protein levels in HT29 and SW707 CRC cells. Moreover, we determined the level of methylation in the 5' flanking region of *HSD17B1 *in primary cancerous and histopathologically unchanged colonic tissues as well as HT29 and SW707 CRC cells treated with 5-dAzaC.

## Methods

### Patient material

Primary colonic adenocarcinoma tissues were collected between June 2009 and June 2010 from fifty two patients who underwent radical colon surgical resection at the Department of General and Colorectal Surgery, Poznań University of Medical Sciences, Poland (Table 1). Histopathologically unchanged colonic mucosa located at least 10-20 cm away from the cancerous lesions was obtained from the same patients. Samples were immediately snap-frozen in liquid nitrogen and stored at −80 º C until RNA/DNA/protein isolation. At the time of surgery, the median and mean age of the patients was 70 years (range 39-85) and 68.36 ± 11.7 years, respectively. Two of the patients exhibited T1 tumour stage, nine of the patients exhibited T2 tumour stage, thirty seven patients exhibited T3 tumour stage and four patients exhibited T4 tumour stage. Written informed consent was obtained from all participating individuals. The procedures of the study were approved by the Local Ethical Committee of Poznań University of Medical Sciences.

**Table 1:** HSD17B1 transcript and protein levels in primary cancerous and histopathologically unchanged tissue samples from patient with CRC.

n = 52		Primary cancerous tissues		Histopathologically unchanged tissues		P^c^
			
		Median (range)	Mean (± SD)	Median (range)	Mean (± SD)	
**Age (Years)**						

<60	10	3.511 (2.109-3.868) ^a^	3.282 ± 0.5921 ^a^	3.415 (2.726-5.107)^a^	3.611 ± 0.6309 ^a^	0.2450^a^
		
		2.634 (1.681-3.218)^b^	2.579 ± 0.5243^b^	2.620 (2.108-3.398)^b^	2.719 ± 0.4843^b^	0.5423^b^

>60	42	3.531 (2.071-5.227) ^a^	3.569 ± 0.6649 ^a^	3.692 (2.790-5.785)^a^	3.834 ± 0.6251^a^	0.1087^a^
		
		2.728 (1.728-4.023)^b^	2.720 ± 0.4702^b^	2.952 (1.364-3.472)^b^	2.835 ± 0.4913^b^	0.1121^b^

**Gender**						

Female	20	3.384 (2.109-4.455) ^a^	3.371 ± 0.5676 ^a^	3.449 (2.726-5.785)^a^	3.719 ± 0.7333^a^	0.2340 ^a^
		
		2.793 (1.681-3.218)^b^	2.666 ± 0.4767^b^	2.811 (2.108-3.472)^b^	2.844 ± 0.4065^b^	0.2119^b^

Male	32	3.571 (2.071-4.653) ^a^	3.557 ± 0.6523 ^a^	3.857(2.790-5.259) ^a^	3.890 ± 0.5855 ^a^	0.0388 ^a^
		
		2.702 (1.728-4.023)^b^	2.713 ± 0.4941^b^	2.962 (1.364-3.449)^b^	2.792 ± 0.5395^b^	0.2832^b^

**CRC localization**						

Proximal colon (cecum to transverse)	20	3.507(2.666-4.455) ^a^	3.427 ± 0.4350 ^a^	3.874 (3.050-5.785) ^a^	4.057 ± 0.7488 ^a^	0.0016 ^a^
		
		2.793 (1.681-3.218)^b^	2.666 ± 0.4767^b^	2.811 (2.108-3.472)^b^	2.844 ± 0.4065^b^	0.0028^b^

Distal colon (splenic flexture to sigmoid)	12	3.214 (2.071-4.313) ^a^	3.218 ± 0.7760 ^a^	3.449 (2.726-4.587) ^a^	3.630 ± 0.6372 ^a^	0.1685 ^a^
		
		2.798 (1.772-3.254)^b^	2.691 ± 0.4777^b^	2.860 (1.364-3.391)^b^	2.619 ± 0.6350^b^	0.7763^b^

Rectum	20	3.544 (2.523-4.653) ^a^	3.657 ± 0.6011 ^a^	3.564 (3.226-4.862) ^a^	3.762 ± 0.500 ^a^	0.8839 ^a^
		
		2.951 (1.728-3.398)^b^	2.815 ± 0.4436^b^	2.988 (2.019-3.383)^b^	2.913 ± 0.3871^b^	0.5019^b^

**Histologic grade**						

G1	4	4.059 (2.362-4.402) ^a^	3.721 ± 0.9467 ^a^	4.431 (3.123-5.259) ^a^	4.311 ± 0.9076 ^a^	0.4026 ^a^
		
		2.926 (2.586-3.040)^b^	2.870 ± 0.2060^b^	2.789 (2.026-3.147)^b^	2.688 ± 0.5370^b^	0.5506^b^

G2	35	3.379 (2.109-4.621) ^a^	3.363 ± 0.5385 ^a^	3.524 (2.726-5.785) ^a^	3.681 ± 0.5619 ^a^	0.0335 ^a^
		
		2.647 (1.681-4.023)^b^	2.649 ± 0.4993^b^	2.933 (2.019-3.472)^b^	2.863 ± 0.4114^b^	0.0659^b^

G3	13	3.875 (2.071-4.839) ^a^	3.807 ± 0.7092 ^a^	4.043 (2.790-5.227) ^a^	4.165 ± 0.7346 ^a^	0.2587 ^a^
		
		2.676 (1.845-3.431)^b^	2.670 ± 0.5098^b^	3.023 (1.364-3.449)^b^	2.763 ± 0.6858^b^	0.5114^b^

The HSD17B1 transcript levels^a ^were standardized by the geometric mean of PBGD and hMRPL19 cDNA and expressed as decimal logarithm of multiples of these cDNA copies in calibrator. ^b ^The amount of western blot-detected proteins was presented as the decimal logarithm HSD17B1 to β-actin band optical density ratio. ^c ^Unpaired, two-tailed *t*-test or U-Mann-Whitney test.

### Antibodies and Reagents

Goat polyclonal (Gp) anti-HSD17B1 antibodies (Ab) (C-18), donkey anti-goat horseradish peroxidase (HRP)-conjugated Ab, anti-actin HRP-conjugated Ab (clone I-19) and anti-Polymerase II (Pol II)-rabbit polyclonal Ab (H-224) were provided by Santa Cruz Biotechnology (Santa Cruz, CA). E1, 5-dAzaC was purchased from Sigma-Aldrich Co. (St. Louis, MO). Goat anti-rabbit HRP conjugated Ab was provided by DAKO (Glostrup, Denmark).

### Cell culture

HT29 colon cancer cells were obtained from the American Type Culture Collection (Rockville, MD) and SW707 CRC cells were kindly provided by the Institute of Immunology and Experimental Therapy of the Polish Academy of Sciences. These cells were cultured in DMEM GibcoBRL (Grand Island, NY) containing 10% heat-inactivated fetal bovine serum (FBS) and 2 mM glutamine. To determine the effect of 5-dAzaC on *HSD17B1 *transcript and protein levels, the HT29 and SW707 cells were cultured for 24 h in phenol red-free DMEM GibcoBRL (Grand Island, NY) supplemented with 10% charcoal-dextran-stripped FBS from Sigma-Aldrich Co. (St. Louis, MO). Cells were then cultured for 6, 12, 24, 36 and 48 h either in the absence or in the presence of 5-dAzaC at concentrations of 0.33, 0.66 and 1.00 μM. These cells were used for total RNA isolation, western blotting, chromatin immunoprecipitation (ChIP) assay, and bisulfite sequencing.

### Reverse transcription and real-time quantitative polymerase chain reaction (RQ-PCR) analysis of the HSD17B1 transcript levels

The total RNA from primary tissues of patients with CRC and from HT29 and SW707 cells was isolated according to the method of Chomczyński and Sacchi (1987) [16]. RNA samples were treated with DNase I, quantified, and reverse-transcribed into cDNA. RQ-PCR was carried out in a Light Cycler real-time PCR detection system from Roche Diagnostics GmbH, (Mannheim, Germany) using SYBR^® ^Green I as detection dye. The target cDNA was quantified by relative quantification method using a calibrator for primary tissue or respective controls for HT29 and SW707 cells. The calibrator was prepared as a cDNA mix from all of the patients' samples and successive dilutions were used to create a standard curve as described in Relative Quantification Manual Roche Diagnostics GmbH, (Mannheim, Germany). For amplification, 1 μl of cDNA solution was added to 9 μl of IQ™ SYBR^® ^Green Supermix Bio-Rad Laboratories Inc. (Hercules, CA) and primers (Additional file 1, Additional file 2). The quantity of transcript of HSD17B1 in each sample was standardized by the geometric mean of porphobilinogen deaminase (PBGD) and human mitochondrial ribosomal protein L19 (hMRPL19) cDNA levels (Additional file 2) [17,18]. The transcript levels in patient tissues were expressed as multiplicity of these cDNA concentrations in the calibrator. In HT29 and SW707 cells, transcript levels were presented as multiplicity of the respective controls.

### Western blotting analysis

Primary tissue from patients with CRC and HT29 and SW707 cells were treated with lysis RIPA buffer, and 40 μg of protein were resuspended in sample buffer and separated on 12% Tris-glycine gel using sodium dodecyl sulfate-polyacrylamide gel electrophoresis (SDS-PAGE). Gel proteins were transferred to a polyvinylidene fluoride (PVDF) membrane, which was blocked with 5% milk in Tris/HCl saline/Tween buffer. Immunodetection of bands was performed with Gp anti-HSD17B1 Ab (C-18), followed by incubation with donkey anti-goat HRP-conjugated Ab. To ensure equal protein loading of the lanes, the membrane was re-stripped and incubated with anti-actin HRP-conjugated Ab (clone I-19). Bands were revealed using ECL kit and Hyperfilm ECL Amersham (Piscataway, NJ). The amounts of HSD17B1 protein was presented as the HSD17B1 to-β-actin band optical density ratio. For HT29 and SW707 cells cultured in the absence of 5-dAzaC, the ratio of HSD17B1 to β-actin was assumed to be 1.

### DNA methylation evaluation by bisulfite sequencing

Genomic DNA was isolated using DNA Mammalian Genomic Purification Kit purchased from Sigma-Aldrich Co. (St. Louis, MO). One μg of genomic DNA was subjected to bisulfite conversion of cytosine to uracil according to EZ DNA Methylation Kit™ procedure from Zymo Research Corporation **(**Orange, CA). The position of CpG islands (chr17: 37 953 426 - 37 954 646) and binding sites of transcription factors located in the 5' flanking region of the promoter was determined by online programs [19-21] (Additional file 1). A DNA fragment containing 31 CpG dinucleotides was amplified from the bisulfite-modified DNA by the primer pair (chr17: 37 953 392-37 953 917) (Additional file 1, Additional file 2) complementary to the bisulfite-DNA modified sequence. PCR amplification was performed by FastStart Taq DNA Polymerase from Roche Diagnostic GmbH (Penzberg, Germany). The PCR products were purified using Agarose Gel DNA Extraction Kit Roche (Mannheim, Germany) with subsequent cloning into pGEM-T Easy Vector System I Promega (Madison, WI) and transformation into TOPO10 *E. coli *strain cells. Plasmid DNA isolated from ten positive bacterial clones was used for commercial sequencing of the cloned fragment of DNA. The results of bisulfite sequencing were assessed and presented using BiQ analyzer software and the Bisulfite sequencing Data Presentation and Compilation (BDPC) web server, respectively [22,23].

### DNA methylation assessment by high resolution melting (HRM) analysis

Methylation levels of three DNA fragments located within the CpG rich region (Additional file 1) were determined by Real Time PCR amplification of bisulfite treated DNA, followed by HRM profile analysis by CFX96™ Real-Time PCR Detection System, Bio*-*Rad Laboratories Inc. (Hercules, CA). For PCR amplification, 1 μl solution of bisulfite treated DNA, from patients or standards, and primers (Additional file 1, Additional file 2) was added to 19 μl of 5 × Hot FIREPol EvaGreen HRM Mix, Solis BioDyne Co. (Tartu, Estonia). Standards of DNA methylation percentage were prepared by mixing in different ratio methylated and non-methylated bisulfite treated DNA from Human Methylated/Non-methylated DNA Set Zymo Research Corp. (Orange, CA). To determine the percentage of methylation, the HRM profiles of patient DNA PCR products were compared with HRM profiles of standard DNA PCR product [24] (Additional file 3). HRM methylation analysis was performed using Precision Melt Analysis™ Software, Bio-Rad Laboratories Inc. (Hercules, CA). Each PCR amplification and HRM profile analysis was performed in triplicate. The methylation for each patient was presented as a percentage of methylation in three amplified fragments located in the CpG rich region of *HSD17B1*.

### ChIP analysis

HT29 and SW707 cells were maintained in phenol red-free DMEM GibcoBRL supplemented with 10% charcoal-dextran-stripped FBS for 6, 12, 24, 36 and 48 h either in the absence or in the presence of 5-dAzaC at a concentration of 1.00 μM. Cells were then fixed by the addition of 270 μL of 37% formaldehyde to 10 mL of culture medium for 10 min at 37 ºC, and harvested. Chromatin from 10^6 ^cells was sheared by sonicator and precleared with salmon sperm DNA-saturated protein G sepharose. The ChIP assay was conducted using Abcam ChIP Kit ab500 [25]. Chromatin was incubated with 2 μg of rabbit polyclonal anti-Pol II Ab (H-224) overnight at 4 ºC. The input and immunoprecipitated DNA were used as templates for Real Time PCR preformed using the primers corresponding to the promoter of the *HSD17B1 *gene (Additional file 1, Additional file 2). The percentage of *HSD17B1 *promoter that was bound with Pol II was calculated based on the differences between C_T _values for input and DNA samples from cells treated or untreated with 5-dAzaC.

### Effect of 5-dAzaC on the conversion of E1 to E2 in HT29 and SW707 cells

To determine the effect of 5-dAzaC on the conversion of E1 to E2, the HT29 and SW707 cells were maintained for 24 h in phenol red-free DMEM GibcoBRL supplemented with 10% charcoal-dextran-stripped FBS from Sigma-Aldrich Co. Cells were then cultured for 36 h either in the absence or in the presence of 5-dAzaC at a concentration of 1.00 μM for HT29 and SW707 cells. These cells were then further cultured either in the absence or the presence of E1 at a concentration of 10^−2 ^mM. After 2, 4, 6, 8, 10 and 12 h, 0.5 ml of the medium was collected and the concentration of E2 was determined by electrochemiluminescence method using Cobas 6000 Roche Diagnostics GmbH (Mannheim, Germany). The results were expressed as E2 concentration in culture medium in nM per μg cell protein.

### Statistical analysis

Data groups for cell lines were assessed by ANOVA to evaluate if there was significance (*P *< 0.05) between the groups. For all experimental groups, which fulfilled the initial criterion, individual comparisons were performed by *post hoc *Tukey test with the assumption of two-tailed distribution and two samples with equal variance at the *P *< 0.05 level. Statistical significance was designated by asterisks in the figures.

The normality of the observed patient data distribution was assessed by Shapiro-Wilk test, and parametric unpaired, two-tailed *t*-test or nonparametric U-Mann-Whitney test was used to compare the mean values. *P *< 0.05 was considered statistically significant. The correlation between mRNA and protein levels was evaluated by Spearman nonparametric correlation coefficient method. The statistical analysis was performed with STATISTICA 6.0 software.

## Results

### HSD17B1 transcript and protein levels in primary cancerous and histopathologically unchanged tissues from patients with CRC

**Figure 1:**
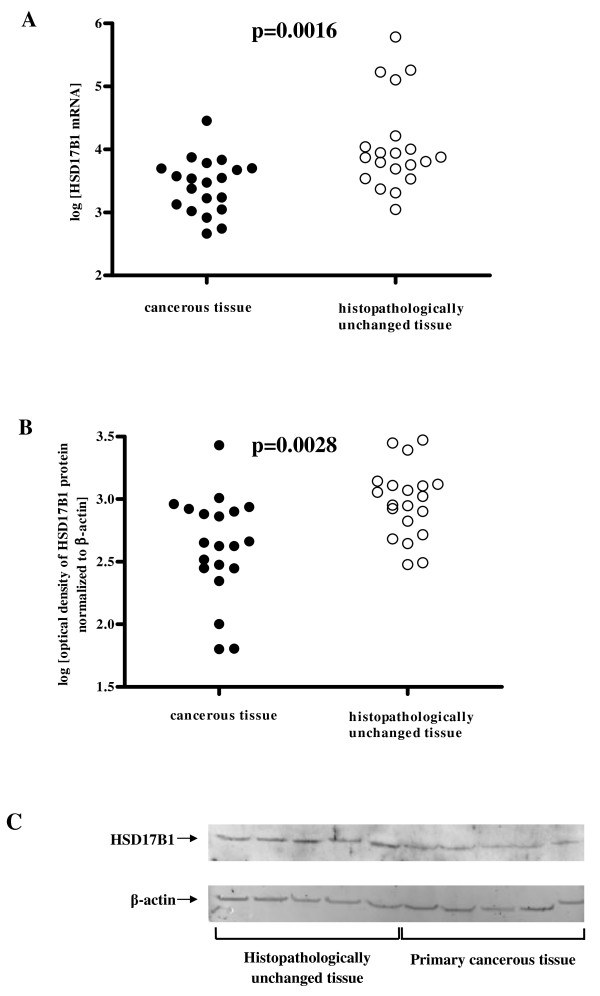
**HSD17B1 transcript (A) and protein (B) levels and representative picture of western blot (C) in primary cancerous and histopathologically unchanged tissues from patients with CRC in the proximal colon**. The cancerous (●) and histopathologically unchanged tissues (○) from twenty patients with CRC in the proximal colon were used for RNA and protein isolation. Total RNA was reverse-transcribed, and cDNAs were investigated by RQ-PCR relative quantification analysis. The HSD17B1 mRNA levels were corrected by the geometric mean of PBGD and hMRPL19 cDNA levels. The amounts of HSD17B1 mRNA were expressed as the decimal logarithm of multiples of these cDNA copies in the calibrator. Proteins were separated by 12% SDS-PAGE, and transferred to a membrane that was then immunoblotted with Gp anti- HSD17B1 Ab and incubated with donkey anti-goat HRP-conjugated Ab. The membrane was then reblotted with anti-actin HRP-conjugated Ab. The amount of western blot-detected HSD17B1 proteins was presented as the decimal logarithm of HSD17B1 to β-actin band optical density ratio. The *p *value was evaluated by unpaired, two-tailed *t*-test.

To compare HSD17B1 transcript and protein levels in cancerous and histopathologically unchanged tissues from patients with CRC we used RQ-PCR and western blotting analysis, respectively. We found significantly lower levels of HSD17B1 transcript (*p *= 0.0016) and protein (*p *= 0.0028) in primary cancerous tissues than in histopathologically unchanged tissues in patients with CRC in the proximal colon (Figure 1A,B,C and Table 1). Moreover, we observed a moderate correlation between mRNA and protein in cancerous (*p *= 0.0502) and histopathologically unchanged tissue (*p *= 0.0722) in the proximal colon. We also observed significantly lower amounts of HSD17B1 transcript (*p *= 0.0335, *p *= 0.0388) and protein (*p *= 0.0659, *p *= 0.2832) in cancerous tissues compared to histopathologically unchanged tissues in patients with G2 histological grade and male patient group, respectively. There were no significant differences in transcript (*p *= 0.1685, *p *= 0.8839) and HSD17B1 protein (*p *= 0.7763, *p *= 0.5019) levels between primary cancerous and histopathologically unchanged tissues in patients with CRC located in the distal colon and rectum (Table 1). We also did not observe differences in HSD17B1 transcript and protein levels between these tissues in the female patient group, different ages, G1/G3 histological grade (Table 1), and different tumour stage (not shown).

### DNA methylation levels in primary cancerous and histopathologically unchanged tissues from patients with CRC

**Figure 2:**
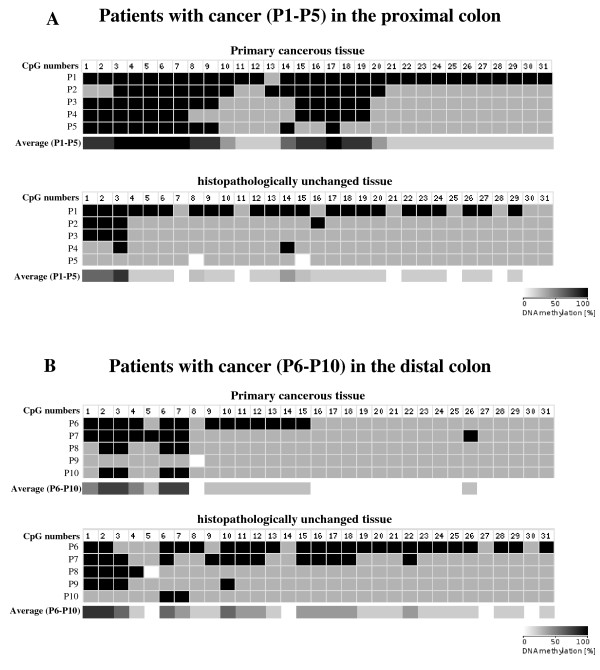
**Bisulfite sequencing of DNA CpG rich region in primary tissue samples from patients with CRC in the proximal (A) and distal (B) colon**. The primary cancerous and histopathologically unchanged tissues from the same patients with cancer in the proximal (P1-P5) and distal colon (P6-P10) were used for genomic DNA isolation followed by bisulfite conversion of cytosine to uracil. The HSD17B1 region containing 31 CpG dinucleotides (chr17: 37 953 392-37 953 917) was then amplified by a pair of primers complementary to the bisulfite-DNA modified sequence (Additional file 1, Additional file 2). The PCR products were purified with subsequent cloning into a plasmid vector. Plasmid DNA isolated from ten positive bacterial clones was used for commercial sequencing. The results of bisulfite sequencing were assessed and presented using BiQ analyzer software and BDPC web server, respectively [22,23]. Black and grey boxes represent methylated and unmethylated CpG dinucleotide, respectively. White boxes correspond to undetermined CpG dinucleotide. The legend with grey scale corresponds to average methylation in (P1-P5) and (P6-P10).

To compare DNA methylation levels in the *HSD17B1 *CpG rich region between DNA from cancerous and histopathologically unchanged tissues, we performed sodium bisulfite DNA sequencing and HRM analysis (Additional file 1, Additional file 2). Bisulfite sequencing was used for preliminary evaluation of DNA methylation changes in randomly selected patients. We observed the same pattern of methylation within all individual clones of each patient. Moreover, this evaluation showed significant differences in DNA methylation levels between cancerous and histopathologically unchanged tissues from five patients with CRC in the proximal colon (Figure 2A). However, we did not observe this difference in five patients with CRC located in the distal colon (Figure 2B) or rectum (not shown). We applied HRM analysis of PCR amplified bisulfite treated DNA to extend the DNA methylation studies in the *HSD17B1 *CpG rich region for cancerous and histopathologically unchanged tissues from all fifty two patients. In patients with CRC located in the proximal colon, we found a significantly higher DNA methylation percentage in cancerous than in histopathologically unchanged tissues (*p *= 0.003) (Table 2). However, there were no significant differences in DNA methylation percentage between cancerous and histopathologically unchanged tissues from patients with CRC located in the distal colon (*p *= 0.7498) and rectum (*p *= 0.9810) (Table 2). We also did not find significant differences in the DNA methylation percentage between these tissues in patients divided based on gender, age, histological grade (Table 2) and tumour stage (not shown).

**Table 2:** The percentage of DNA methylation in CpG rich regions in primary cancerous and histopathologically unchanged tissue samples from patients with CRC.

**n = 52**		**Primary cancerous tissues**		**Histopathologically unchanged tissues**		**p^a^**
		**Median (range)**	**Mean (± SD)**	**Median (range)**	**Mean (± SD)**	
**Age**						
<60	10	21.05 (12.89-25.79)	20.66 ± 4.205	19.47 (13.16-28.42)	19.32 ± 4.875	0.5181
>60	42	20.00 (4.211-33.16)	20.91 ± 7.074	18.95 (2.105-36.21)	18.42 ± 6.779	0.1076
**Gender**						
Female	20	24.21 (16.84-33.16)	24.42 ± 4.516	23.68 (13.68-28.42)	21.75 ± 4.499	0.1155
Male	32	18.95 (4.211-32.11)	19.51 ± 7.255	17.37 (2.105-36.21)	18.43 ± 7.206	0.5594
**CRC localization**						
Proximal colon (cecum to transverse)	20	24.21 ( 13.68-33.16)	23.54 ± 5.793	17.63 ( 2.105435.79)	17.61 ± 6.050	0.0030
Distal colon (splenic flexture to sigmoid)	12	19.47 (4.211-28.95)	19.69 ± 7.159	18.95 (10.53-25.26)	18.85 ± 4.747	0.7498
Rectum	20	19.47 (6.316-28.42)	19.36 ± 6.292	19.47 (4.737-36.21)	19.30 ± 7.851	0.9810
**Histologic grade**						
G1	4	21.32 (13.68-28.42)	21.18 ± 8.067	17.89 (2.105-27.89)	16.45 ± 10.97	0.5126
G2	35	20.00 (10.53-30.53)	21.17 ± 5.293	19.47 (4.737-28.42)	18.93 ± 5.884	0.0996
G3	13	21.84 (4.211-33.16)	20.21 ± 9.397	15.53 (12.63-36.21)	18.11 ± 6.727	0.4021

The primary cancerous and histopathologically unchanged tissue samples from the same patients were used for genomic DNA isolation, followed by bisulfite conversion of cytosine to uracil. The three DNA fragments of the CpG rich region were then amplified by three pairs of primers complementary to the bisulfite-DNA modified sequence (Additional file 1, Additional file 2). To determine the percentage of methylation, the HRM profiles of the patients' DNA PCR products were compared to HRM profiles of the prepared standard PCR products. DNA methylation for each patient was presented as a percentage of methylation in three DNA amplified fragments located in the CpG rich region (Additional file 1). ^a ^Unpaired, two-tailed *t*-test.

### 5-dAzaC effects on HSD17B1 transcript and protein contents in HT29 and SW707 colorectal cancer cells

**Figure 3:**
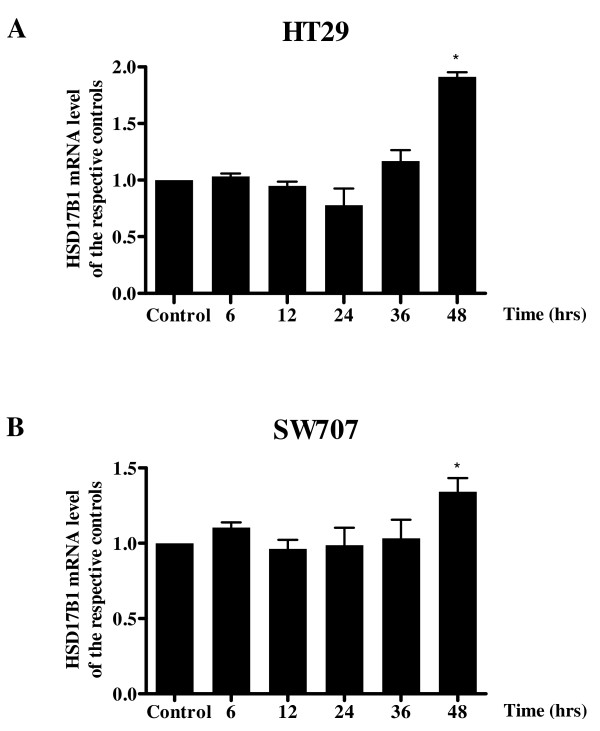
**5-dAzaC up-regulates HSD17B1 transcript levels in HT29 (A) and SW707 (B) colorectal cancer cells**. HT29 and SW707 cells were cultured in phenol red-free DMEM for 6, 12, 24, 36 and 48 h either in the absence or in the presence of 5-dAzaC at a concentration of 1.00 μM. After incubation the cells were used for total RNA isolation and reverse transcription. The HSD17B1 cDNA levels were determined by RQ-PCR relative quantification analysis. RQ-PCR results were standardized by the geometric mean of PBGD and hMRPL19 cDNA levels. HSD17B1 cDNA levels are expressed as a multiplicity of the respective controls. Each sample was determined in triplicate and results are presented as the mean ± SE from three experiments *P < 0.05.

**Figure 4:**
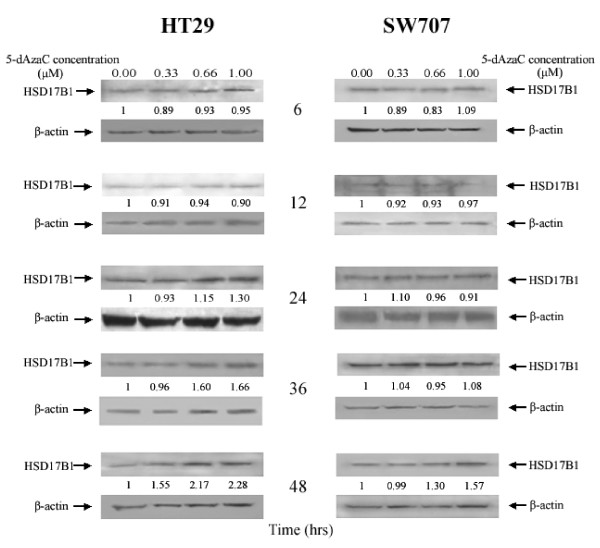
**5-dAzaC effect on HSD17B1 protein levels in HT29 and SW707 colorectal cancer cells**. Cells were cultured in phenol red free DMEM media for 6, 12, 24, 36 and 48 h either in the absence or in the presence of 5-dAzaC at a concentration of 0.33, 0.66 and 1.00 μM. The cell protein was separated by 12% SDS-PAGE, and transferred to a membrane that was then immunoblotted with Gp anti-HSD17B1 Ab and incubated with donkey anti-goat HRP-conjugated Ab. The membrane was then reblotted with anti β-actin HRP-conjugated Ab. The band densitometry readings were normalized to β-actin loading control. The ratio HSD17B1 to β-actin for control was assumed to be 1.

We observed that 5-dAzaC resulted in a progressive increase in HSD17B1transcript levels in HT29 and SW707 cells (Figure 3A,B). For HT29 cells, we found approximately a 1.91- fold significant increase in HSD17B1 transcript levels at 48 h of incubation (Figure 3A). There was also an approximately 1.35-fold significant increase in HSD17B1 mRNA in SW707 cells at 48 h of incubation (Figure 3B). These alterations in HSD17B1 transcript levels in both HT29 and SW707 cells were associated with increased HSD17B1 protein levels (Figure 4). Densitometric analysis of western blotting bands indicated a gradual increase of HSD17B1 protein levels in a dose dependent manner at 24, 36, and 48 h of incubation for HT29 cells, and at 48 h for SW707 cells (Figure 4). Incubation of HT29 cells with 5-dAzaC at a concentration of 1.00 μM for 48 h resulted in a 2.28-fold increase in HSD17B1 protein contents (Figure 4). There was also an approximately 1.57-fold increase in HSD17B1 protein contents in SW707 cells incubated with 5-dAzaC at a concentration of 1.00 μM for 48 h as compared with the respective control (Figure 4).

### 5-dAzaC induced transcription of *HSD17B1 *via DNA demethylation in the CpG rich region and increased the binding of Pol II to the promoter in HT29 colon cancer cells

**Figure 5:**
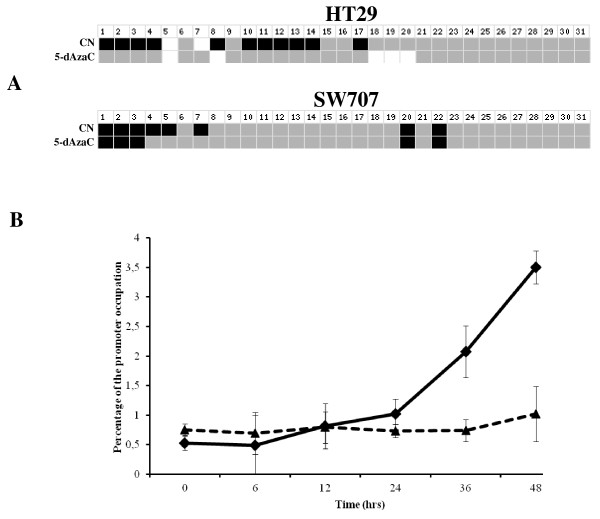
**Bisulfite sequencing of the CpG rich region fragment (A) and binding of Pol II to *HSD17B1 *promoter (B) in HT29 and SW707 colorectal cancer cells treated with 5-dAzaC**. HT29 and SW707 cells were incubated for 48 h either in the absence or in the presence of 5-dAzaC (1.00 μM). The cells were then used for genomic DNA isolation followed by bisulfite conversion of cytosine to uracil. The CpG rich region containing 31 CpG dinucleotides (chr17: 37 953 392-37 953 917) was then amplified by a pair of primers complementary to the bisulfite-DNA modified sequence (Additional file 1, Additional file 2). The PCR products were purified with subsequent cloning into a plasmid vector. Plasmid DNA isolated from ten positive bacterial clones was used for commercial sequencing. The results of bisulfite sequencing were assessed and presented using BiQ analyzer software and BDPC web server, respectively [22,23]. Black and grey boxes represent methylated and unmethylated CpG dinucleotide, respectively. White boxes correspond to undetermined CpG dinucleotide. For the ChIP assay, HT29 and SW707 cells were incubated for 0, 6, 12, 24, 36, and 48 h either in the absence or in the presence of 5-dAzaC (1.00 μM). After incubation, cells were used for ChIP analysis with anti-Pol II Ab. RQ-PCR was carried out by pairs of primers complementary to the *HSD17B1 *promoter for the HT29 (-◆-) and SW707 (...▲...) cell lines (Additional file 1, Additional file 2). Data are expressed as a percentage of the *HSD17B1 *promoter occupied by Pol II. The results are presented as the mean ± SE from three independent experiments.

In order to assess the effect of 5-dAzaC on DNA demethylation and Pol II binding to the promoter we used bisulfite DNA sequencing and the ChIP assay, respectively (Additional file 1). We noticed the same pattern of methylation within all analysed clones. Moreover, we observed significant DNA demethylation in the CpG rich region in HT29 cells cultured for 48 h in the presence of 5-dAzaC (Figure 5A). Incubation with 5-dAzaC at a concentration of 1.00 μM for 6, 12, 24, 36, and 48 h caused an approximately 1.56 to 6.70-fold increase in the percentage of promoter occupancy by Pol II in HT29 colon cancer cells (Figure 5B). However, in SW707 CRC cells treated with 5-dAzaC at a concentration of 1.00 μM, we observed slight DNA demethylation and slight increased occupancy percentage of the promoter by Pol II (Figure 5A,B).

### 5-dAzaC increased the conversion of E1 to E2 in HT29 and SW707 colorectal cancer cells

**Figure 6:**
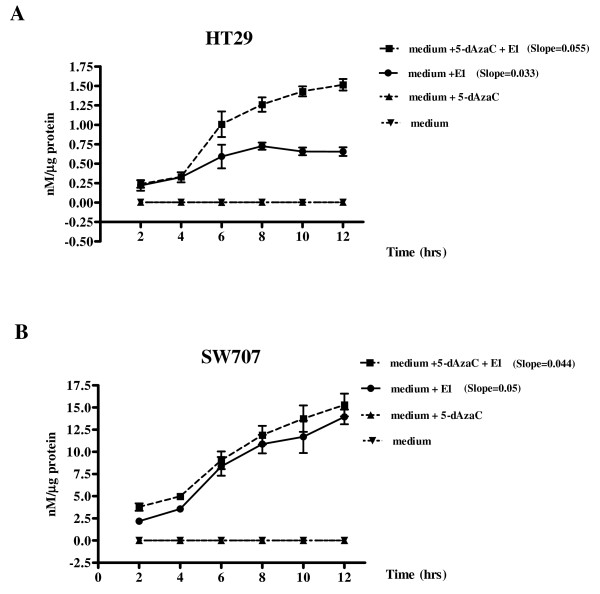
**Effect of 5-dAzaC in conversion of E1 to E2 in HT29 (A) and SW707 (B) colorectal cancer cells**. Cells were cultured in phenol red-free DMEM for 36 h either in the absence or in the presence of 5-dAzaC at a concentration of 1.00 μM for HT29 and SW707 cells. These cells were then further cultured either in the absence or the presence of E1 at a concentration of 10^-2 ^mM. After 2, 4, 6, 8, 10 and 12 h, 0.5 ml of the medium was collected and the concentration of E2 was determined by electrochemiluminescence method. The results were expressed as E2 concentration in culture medium as nM per μg cell proteins. Each sample was determined in triplicate and results are presented as the mean ± SE from three experiments. The slope values were calculated according to nonlinear regression data fit from the log of Y axis values.

5-dAzaC caused a progressive increase in the conversion of E1 to E2 in a time dependent manner in 5-dAzaC pretreated HT29 cells at 6, 8, 10 and 12 h of incubation (Figure 6A). Incubation of 5-dAzaC pretreated HT29 cells with E1 for 12 h resulted in a 3.0-fold increase in E2 levels as compared to 5-dAzaC untreated cells (Figure 6A). There was also a slight, 1.2-fold, increase in E2 concentration in 5-dAzaC pretreated SW707 cells at 12 h of incubation as compared with the respective control (Figure 6B).

## Discussion

There are many environmental, dietary, lifestyle, behavioral, genetic and epigenetic factors that have been considered as risk factors for developing CRC [26-30]. Malignant transformation of colonic mucosa to adenoma and adenocarcinoma is associated with the accumulation of mutations in various oncogenes, tumour suppressor genes (TSGs) and chromosome, and microsatellite instability [29,31]. Alternatively, the epigenetic mechanism has also been considered as a strong contributor to CRC incidence. Aberrant methylation of DNA may mimic DNA mutation, leading to transcriptional silencing of TSGs and chromosome instability [15].

Accessibility of E2 is another significant factor contributing to the development of CRC. The randomized clinical trial conducted by the Women's Health Institute showed that treatment of postmenopausal women with E2 plus progestin was associated with a decreased risk of CRC [32]. In contrast, treatment of these women with E2 alone exhibited a rather slight increased risk of CRC [32]. However, the importance of E2 in the prevention of large bowel tumorigenesis has been shown in other epidemiological studies [3,5,33]. E2 exerts an apoptotic effect in CRC cells mainly via interaction with the estrogen receptor (ER) [34-36]. This apoptotic effect of E2 in CRC can be inhibited by DNA methylation and silencing of transcription of the *ESR2 *gene encoding ERβ [35,37]. Papaxoinis et al. [38] showed that *ESR2 *expression is higher in normal epithelium of the proximal colon as compared to the distal colon. This indicated that epigenetic silencing of *ESR2 *appearing in 90% of CRC cases could mainly be associated with carcinogenesis in the proximal colon [39]. Moreover, the cohort studies indicated that, in postmenopausal women, CRC more frequently developed in the proximal colon than in the distal colon [40,41]. These findings may indicate that the proximal colon may be the major bowel region, compared to the distal colon, in which E2 may mainly exert its anticancer action [42]. Fiorelli et al. (2002) showed the involvement of HSD17B1 in the conversion of E1 to E2 in human colon cancer cells [43]. This may suggest that the extragonadal production of E2 from E1, especially in the large bowel, may be critical in CRC incidence in postmenopausal women and in men.

During CRC development, epithelial cells acquire peculiar features which enable them to grow aberrantly, ignore growth inhibition, and resist death signals [44]. We found a significant reduction of HSD17B1 transcript and protein levels in CRC in the proximal colon. However, these differences were not found in CRC in the distal colon and rectum. The decreased expression of *HSD17B1 *was associated with significantly increased DNA methylation in the CpG rich region located in the 5' flanking region of the *HSD17B1 *gene in CRC in the proximal colon but not in the distal colon and rectum.

Abnormalities in DNA methylation are observed from the early stages of lesions in aberrant crypt foci and hyperplastic polyps considered to be CRC incidence risk factors [45,46]. To date, transcriptional silencing by DNA methylation has been demonstrated in *hMLH1, PDZ and LIM domain 2, UDP-glucuronosyltransferase 1A1*, *HOXB13*, *CXCL12 *and other genes in CRC [31,47-50]. Moreover, there is a distinction between tumorigenesis associated with DNA methylation and gene expression when comparing the proximal colon to the distal colon and rectum [30,51]. Carcinogenesis in the proximal colon is mainly associated with epigenetic aberrations, whereas cancer incidence in the distal colon and rectum is mainly linked to the accumulation of genetic mutations [51]. Glebov et al. (2003) used microarray analysis to demonstrate that more than 1000 genes are expressed differently in CRC depending on its location in either the ascending or descending colon [52]. Toyota et al. (1999) characterized the CpG island methylator phenotype (CIMP) in CRC [53]. CRC with CIMP-high develops more frequently in the proximal colon, while CIMP-low or CIMP-negative is likely to be found in the distal colon [30,51]. Hypermethylation of the promoter region of *hMLH1*, encoding a component of the DNA mismatch repair system, was also more frequently found in CRC in the proximal colon than in the distal colon [31].

In order to determine the role of methylation of the CpG rich region in *HSD17B1 *expression, we treated HT29 and SW707 cells with 5-dAzaC. We observed DNA demethylation within the CpG rich region (chr17: 37 953 426-37 954 646) (Additional file 1), which was associated with an increase in Pol II binding to the promoter and increased the HSD17B1 transcript and protein levels in HT29 cells. However, 5-dAzaC treatment caused lower DNA demethylation and binding of Pol II to promoter in SW707 cells than in HT29 cells. These differences between HT29 and SW707 cells might be due to their distinct regions of origin from the large bowel: SW707 cells were derived from the rectum, whereas HT29 originated from the colon [54,55].

Data from the Encyclopedia of DNA elements project showed that the analyzed CpG rich region upstream of *HSD17B1 *(chr17: 37 953 426-37 954 646) is able to bind various transcription factors. Moreover, the enrichment of histone modification and DNAse I hypersensitivity of that region suggest promoter or enhancer activity [56]. Lornicz et al. (2004) showed that DNA methylation in intragenic and intergenic CpG rich regions may convert euchromatin to heterochromatin and lead to reduced Pol II action [57]. Based on this observation, we hypothesize that hypermethylation of the 5' flanking region of the *HSD17B1 *gene might inhibit the binding of transcription factors and decrease *HSD17B1 *expression in CRC.

5-dAzaC has been considered in the treatment of CRC. Genome-wide studies, including promoter methylation and expression microarray analysis, revealed a number of genes that are up-regulated in SW116 and Colo-320 CRC cells treated with 5-dAzaC [58]. This DNMTs inhibitor also results in DNA demethylation and induces the expression of *PDZ and LIM domain 2*, *UDP-glucuronosyltransferase 1A1*, *insulin-like growth factor binding protein 7*, and *NGX6 *genes in CRC cells [47,48,59,60]. Lin et al. (2008) demonstrated that 5-dAzaC induced apoptosis and inhibited the growth, migration and invasion of colon cancer cells [60].

## Conclusion

Our results showed increased DNA methylation in the 5' flanking region of *HSD17B1 *in CRC from the proximal colon as compared to normal colonic epithelium. These epigenetic differences were associated with a significant reduction of *HSD17B1 *transcript and protein levels in cancer located in the proximal colon. We also found that 5-dAzaC, an inhibitor of DNMTs, induced the demethylation of the 5' flanking region of *HSD17B1*, leading to increased transcript and protein levels in HT29 colon cancer cells, which contributed to the increase in E2 formation.

Although we presented that *HSD17B1 *expression in CRC can be epigenetically down-regulated, further studies are required to assess the concentration of endogenous E2 in normal colonic tissue and the role of endogenous E2 in the prevention of carcinogenesis.

## Competing interests

The authors declare that they have no competing interests.

## Authors' contributions

AAR contributed to designing the study, acquisition of data, analysis and interpretation of data, and in writing the manuscript. KH participated in the acquisition and interpretation of the data. As Principal Investigators, PK and JPP were involved in the intellectual and experimental programming of the study, the interpretation of data, and writing the manuscript. All authors read and approved the final manuscript.

## Pre-publication history

The pre-publication history for this paper can be accessed here:

http://www.biomedcentral.com/1471-2407/11/522/prepub

## Supplementary Material

Additional file 1 **Organization of the *HSD17B1 *gene (A), the positions of CpG-rich regions and promoter**. Click here for file

Additional file 2 **Primer sequences**. Click here for file

Additional file 3 **DNA methylation assessment by HRM analysis**. Click here for file
