# Supplementary material for: Decreased expression of 17β-hydroxysteroid dehydrogenase type 1 is associated with DNA hypermethylation in colorectal cancer located in the proximal colon
Source: BMC Cancer. 2011 Dec 19;11:522. doi: 10.1186/1471-2407-11-522 (PMC3280200; doi:10.1186/1471-2407-11-522)
Supplement: Additional file 1 — Organization of the HSD17B1 gene (A), the positions of CpG-rich regions and promoter. [file 1471-2407-11-522-S1.PDF]

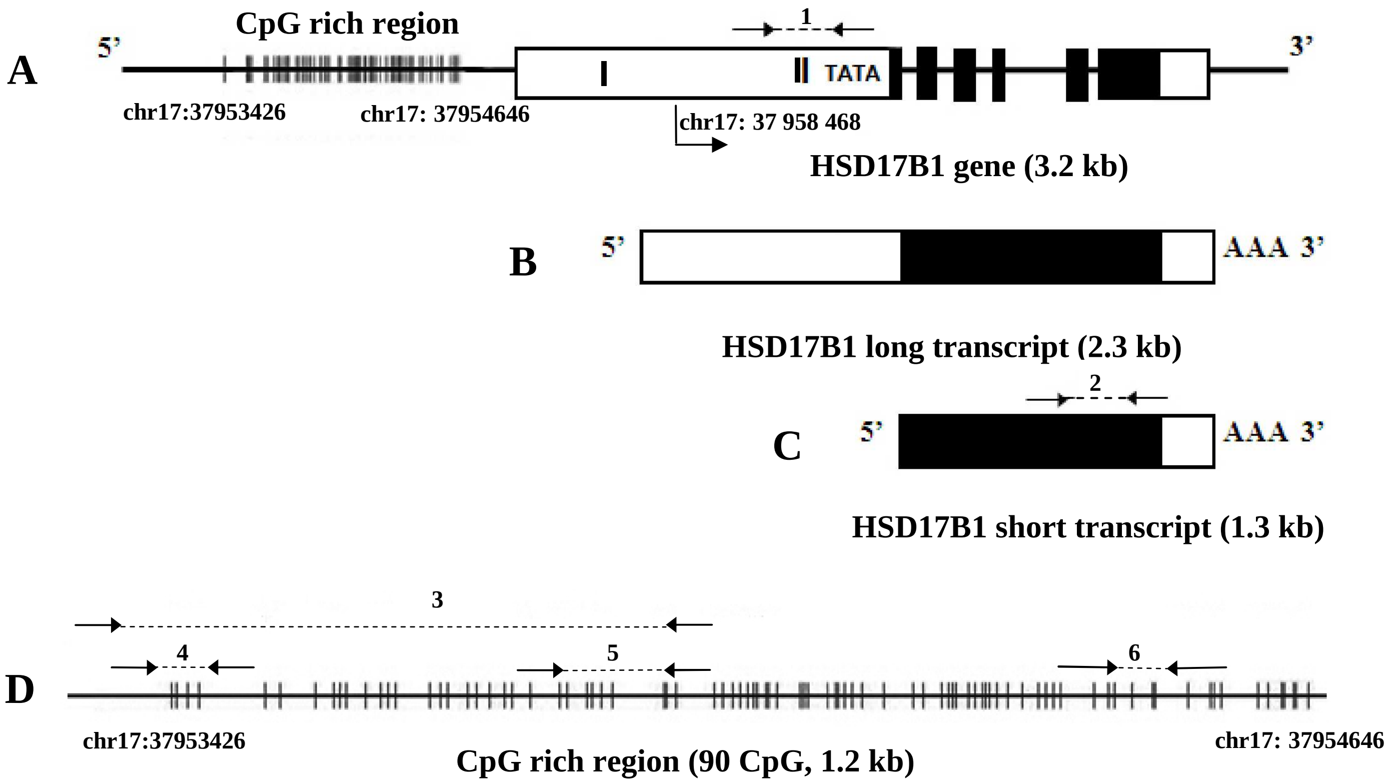

Additional file 1. Organization of the *HSD17B1* gene (A), the positions of CpG-rich regions and promoter.

The *HSD17B1 gene* (chr17: 37 957 510 - 37 960 757, NC_000017) **(A)** is composed of six exons separated by introns (lines). The white and black boxes correspond to the untranslated or translated region of exons, respectively. The promoter II, including the TATA sequence, occupies 100 nucleotides that can be found within the short HSD17B1 transcript (**C**) (chr17: 37 958 368 - 37 958 468)[10-13]. The 90 CpG-rich regions **(D)** were localized on chromosome 17:37 953 426 – 37 954 646. Arrows correspond to the position of primers used for the ChiP assays for promoter (1), for RQ-PCR analysis HSD17B1 transcript (2), and primer localization for bisulfite sequencing within the CpG-rich regions (3). The numbers from 4 to 6 in the CpG-rich regions **(D)** represent the position of the primers used to assess DNA methylation levels by HRM analysis (Additional file 2).
