# Supplementary material for: Decreased expression of 17β-hydroxysteroid dehydrogenase type 1 is associated with DNA hypermethylation in colorectal cancer located in the proximal colon
Source: BMC Cancer. 2011 Dec 19;11:522. doi: 10.1186/1471-2407-11-522 (PMC3280200; doi:10.1186/1471-2407-11-522)
Supplement: Additional file 2 — Primer sequences. [file 1471-2407-11-522-S2.PDF]

Additional file 2. Primer sequences

Gene	Sequence (5’-3’)	UCSC position (NCBI36/hg18)	Product size (bp)
HSD17B1 transcript	TGAGGAGGTGGCGGAGGTCTTC<br>CGCTCGGTGGTGAAGTAG	ch17:37 960 111- 37 960 271	75
ChIP HSD17B1 promoter II	TGGGAGACACAACAAGGGGT<br>CCGATGCCCGAGGAACAGC	chr17: 37 958 383- 37 958 524	142
PBDG	GCCAAGGACCAGGACATC<br>TCAGGTACAGTTGCCCATC	chr11:118 468 348-118 468 864	160
hMRPL19	ACTTTATAATCCTCGGGTC<br>ACTTTCAGCTCATTAACAG	chr2:75 735 389- 75 735 705	171
**Primers for bisulfite and HRM methylation analysis of CpG rich region (Additional file 1)**			
3	TATTTGTTGATTTGGTTGTTGAAGT<br>GTAGTATTTTGAGGAGGTGGTGAAG	chr17: 37 953 392-37 953 917	526
4	TGTTGATTTGGTTGTTGAAGTGTTG<br>GGTAGTTTTAGATGGGTTTGGGAGG	chr17: 37 953 396-37 953 551	156
5	GTTTTTATGTAGAAGGTGTTGGG<br>GTAGTATTTTGAGGAGGTGGTGAAG	chr17:37 953 774-37 953 917	144
6	TGGGATGGGAGATTTTGAGTTTAG<br>TTTTTTTTGTTTTTTGGGTTTGTG	chr17:37 954 206-37 954 307	101

ChIP - chromatin immunoprecipitation, primer localization was presented in Additional file 1, Online supplementary data. HRM-high resolution melting
