# Supplementary material for: Decreased expression of 17β-hydroxysteroid dehydrogenase type 1 is associated with DNA hypermethylation in colorectal cancer located in the proximal colon
Source: BMC Cancer. 2011 Dec 19;11:522. doi: 10.1186/1471-2407-11-522 (PMC3280200; doi:10.1186/1471-2407-11-522)
Supplement: Additional file 3 — DNA methylation assessment by HRM analysis. [file 1471-2407-11-522-S3.PDF]

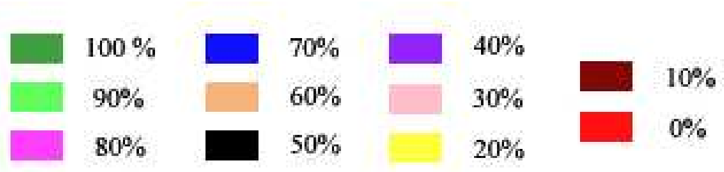

Standards

A

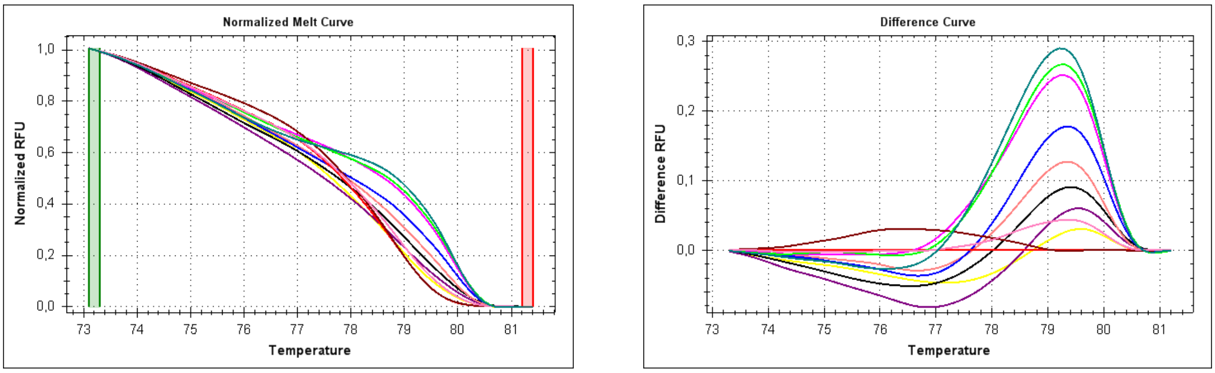

Patients

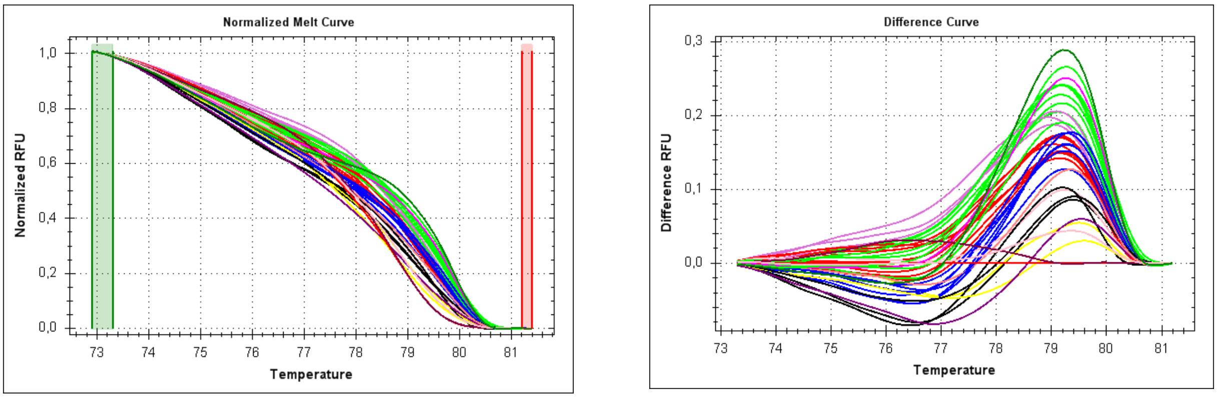

Standards

B

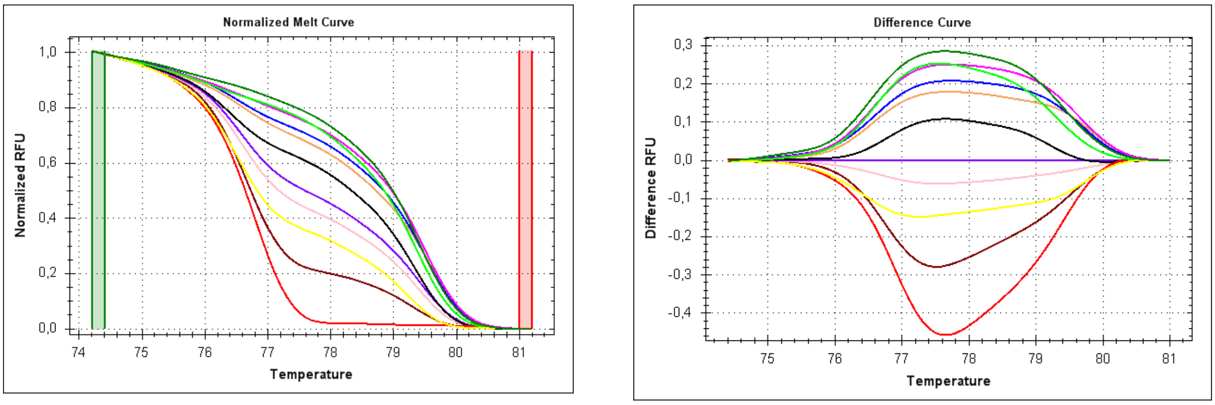

Patients

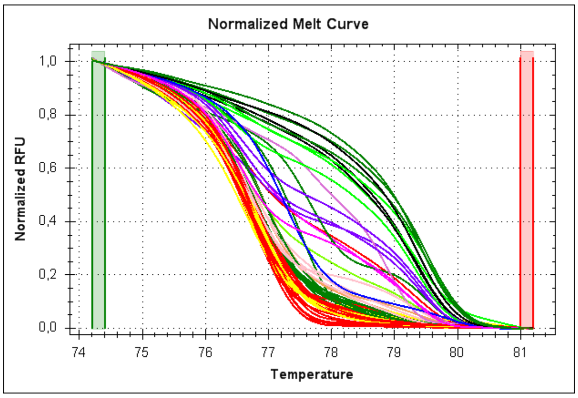

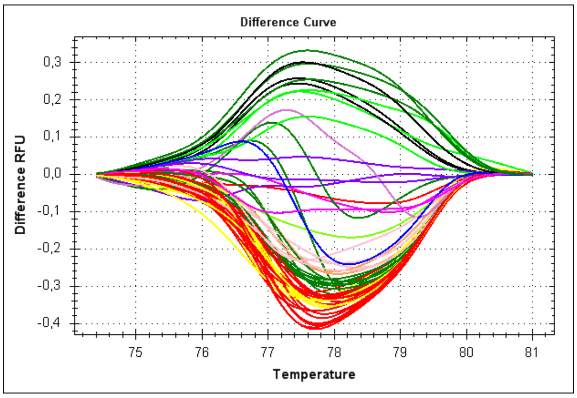

Standards

C

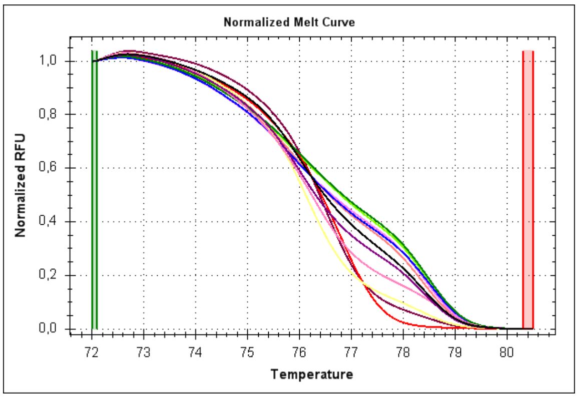

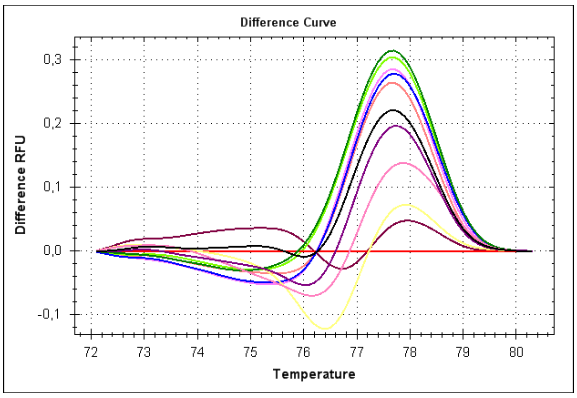

Patients

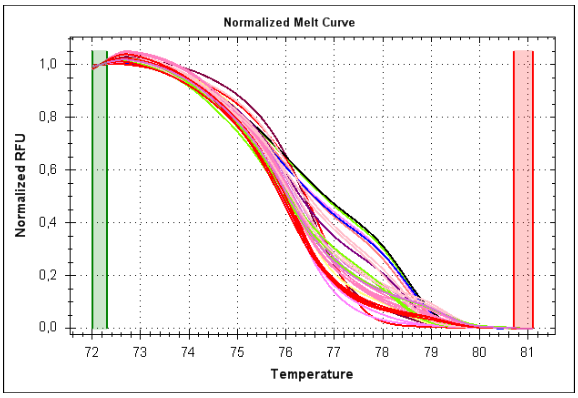

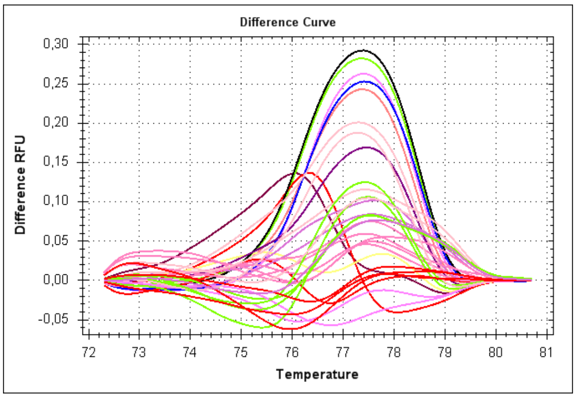

Additional file 3. DNA methylation assessment by HRM analysis.

Methylation percentage of three DNA fragments within the CpG rich region (Additional file 1) was determined by Real Time PCR amplification of bisulfite treated standard and patient DNA, followed by comparison of their HRM profiles. DNA standards were prepared by mixing different ratios of methylated and non-methylated bisulfite treated DNA. HRM methylation analysis was performed using Precision Melt Analysis™ Software, Bio-Rad Laboratories Inc. (Hercules, CA). Each PCR amplification and HRM profile analysis was performed in triplicate. A, B and C represent HRM profiles of standard and patient DNA PCR product for primers numbered 4, 5 and 6 (Additional file 1).
